# Supplementary material for: Unravelling potential virulence factor candidates in Xanthomonas citri. subsp. citri by secretome analysis
Source: PeerJ. 2016 Feb 23;4:e1734. doi: 10.7717/peerj.1734 (PMC4768671; doi:10.7717/peerj.1734)
Supplement: Table S1 [file peerj-04-1734-s001.docx]

**Supplemental table 1**: Other proteins detected

| Gene  ID | GeneName | Product | Cat. | NCBIID | Strains (culture media) | | | | SP | PIP |
| --- | --- | --- | --- | --- | --- | --- | --- | --- | --- | --- |
|  |  |  |  |  | ∆*hrpB4*(NB) | *Xac*(NB) | ∆*hrpB4*(XAM1) | *Xac*(XAM1) |  |  |
| XAC0753 |  | Hypothetical protein | VIII | 1154824 | + | + | - | - | Y | N |
| XAC0542 | *groEL* | Molecular chaperone GroEL | III | 1154613 | + | + | - | - | N | N |
| XAC3514 |  | Serine Protease | III | 1157585 | + | + | - | - | Y | N |
| XAC1012 | *mopB* | OmpA-OmpF porin, OOP family | IV | 1155083 | + | + | - | - | Y | N |
| XAC4199 |  | Polyvinyl alcohol dehydrogenase | I | 1158270 | + | - | + | + | N* | N |
| XAC0868 |  | Hypothetical protein | VIII | 1154939 | + | + | + | + | Y | N |
| XAC2763 |  | Extracellular protease | III | 1156834 | + | - | - | + | Y | N |
| XAC3354 | *ompW* | Outer membrane protein W | IV | 1157425 | + | - | - | + | Y | N |
| XAC0957 | *tuf* | Elongation factor Tu | III | 1155028 | - | + | - | + | N | N |
| XAC2992 | *argC* | Endoproteinase ArgC | III | 1157063 | + | + | - | + | Y | N |
| XAC0798 | *amy* | Alpha-amylase | I | 1154869 | + | - | - | - | Y | N |
| XAC3545 |  | Serine protease | III | 1157616 | + | - | - | - | Y | N |
| XAC1349 |  | Serine protease | III | 1155420 | + | - | - | - | Y | N |
| XAC1550 | *fkpA* | FKBP-type peptidyl-prolyl cis-trans isomerase | III | 1155621 | + | - | - | - | N* | N |

Gene_ID and Cat (primary category): according to da Silva (da Silva et al., 2002); Product: according to Kegg(Ogata et al., 1999); SP – Signal Peptide; Y – Yes; N – No; N* - Secreted by non-classical pathways.
